# Personal Protective Equipment: Attitudes and Behaviors Among Nurses at a Single University Medical Center

**DOI:** 10.7759/cureus.20265

**Published:** 2021-12-08

**Authors:** Samantha Shwe, Aditi A Sharma, Patrick K Lee

**Affiliations:** 1 Dermatology, University of California, Irvine, Irvine, USA; 2 Dermatology, University of California San Francisco, San Francisco, USA

**Keywords:** occupational health, healthcare workers, nurses, covid-19, ppe, personal protective equipment

## Abstract

Introduction: Healthcare workers are at an increased risk of infectious disease transmission through occupational exposure. Despite this, rates of personal protective equipment (PPE) use vary among healthcare personnel. This cross-sectional study surveyed healthcare workers at a single academic center to determine how the coronavirus disease 2019 (COVID-19) pandemic affected the perceptions and behaviors of PPE usage.

Methods: An anonymous online survey through the SurveyMonkey® tool (Zendesk Inc., San Francisco, California) was sent to the University of California, Irvine, Medical Center department listserv of nurses on March 20, 2021, and was closed on June 20, 2021.

Results: Of 311 respondents, 23% admitted to suffering a splash injury to the face during a procedure. When compared to one year ago prior to the COVID-19 pandemic, PPE was more important (93% vs. 80%) and more frequently used (80% vs. 54%) by respondents. The recent COVID-19 pandemic had the strongest impact on increasing respondents’ perception of the importance of PPE (44%).

Conclusion: The COVID-19 pandemic positively impacted rates of PPE usage and perceptions of the importance of PPE among healthcare workers at a single academic institution. Implementing clear and effective education and training programs, ensuring adequate access to protective gear, and promoting a positive safety climate can help improve adherence to safety protocols and appropriate use of PPE.

## Introduction

Healthcare workers are at risk of blood-borne infection from occupational exposure to body fluids and needlestick injuries. Multiple cases documenting Hepatitis B, Hepatitis C, and HIV transmission from direct contamination of the mucous membranes have been reported in the literature [1-6]. Personal protective equipment (PPE) is defined by the Occupational Safety and Health Administration (OSHA) as “specialized clothing or equipment worn to minimize exposure to a variety of hazards” [7]. This includes, but is not limited to, eye protection, mask, gloves, gown, and shoe covers. Guidelines for the use and selection of PPE in healthcare settings have been established by the Centers for Disease Control (CDC) and are based on the type of precautions (i.e., standard, contact, droplet, and airborne) used in context [8].

Nurses are often the first member of the healthcare personnel team to encounter a patient and face a high risk of exposure to blood-borne infections without effective use of PPE. Despite regulations for education and training on the appropriate and correct use of PPE for all healthcare workers, studies have shown that the incidence and prevalence of body fluid contamination injuries across various specialties are not uncommon [3,4,9].

Moreover, the coronavirus disease 2019 (COVID-19) pandemic has had significant implications on the perceptions of and use of PPE, both for healthcare workers and the general public. An international survey of availability and use of PPE among healthcare workers caring for COVID-19 patients in the ICU reported widespread shortages, frequent reuse of, and adverse effects related to PPE [10]. Shortages of PPE supply and inability to meet the demands of rising COVID-19 hospitalizations required rationing of essential PPE gear, including N95 respirators, surgical masks, and face masks. This shortage demanded that healthcare workers understand the indications for various components of PPE with respect to different levels of precautions, in addition to correctly wearing PPE to prevent the spread of infection. Limited access to PPE in the context of a worsening pandemic placed greater emphasis on the importance of PPE use and compliance with safety and infection prevention measures.

The purpose of this cross-sectional study was to assess how attitudes and behaviors regarding PPE use changed because of the COVID-19 pandemic. We hypothesized that the COVID-19 pandemic would influence healthcare workers’ perception of the importance of PPE and the frequency of PPE usage. Specifically, we predict that healthcare workers will regard PPE as more important and use PPE more frequently when compared to a year prior to survey dissemination and that these changes will persist even after the pandemic resolves.

## Materials and methods

The study protocol received Institutional Review Board exemption from the University of California (UC) Irvine Human Research Protections Program. An anonymous online survey through the SurveyMonkey® tool (Zendesk Inc., San Francisco, California) was sent to the UC Irvine Medical Center department listserv of nurses on March 20, 2021, and was closed on June 20, 2021. Dissemination occurred approximately one year after the WHO declared COVID-19 to be a global pandemic. Unit-specific listservs were used to disseminate to nurses throughout multiple departments in the UC Irvine health system. 

The survey consisted of 17 questions pertaining to demographics, attitudes toward PPE, workplace hygiene practices, and PPE usage behaviors before and after the COVID-19 pandemic. Questions on workplace hygiene practices included frequency of hand sanitation, cleaning shared spaces, and cleaning personal items. PPE usage behaviors included wearing gloves, gown, shoe coverings, bouffant cap, face shield or mask with eye protection, and pulling back hair. Questions were also asked on how highly respondents regarded the importance of PPE before and after the pandemic began and identified major influences of their perception. Multiple-choice responses for behavioral questions included “always (100%),” “often (50-99%),” “sometimes (1-49%),” and “never (0%).”

## Results

A total of 311 responses were collected from the survey. The average and median age of respondents was 39 and 38 years, respectively, (range 18 - 67 years); 76.5% were female and 23.5% were male (Table 1). Questions regarding perceptions and usage of PPE compared responses at the time of survey completion to one year ago, pre-pandemic (Table 2). A greater number of respondents (93%) perceived PPE to be “very important” when compared to before the pandemic (80%). Similarly, 80% reported “always” using PPE during a medical procedure with potential bloodborne exposure at the time of survey completion compared to 54% before the pandemic. The major influence on respondents’ perception of the importance of wearing PPE during procedures was the “recent pandemic” (44.5%) followed by “training/educational program” (34%). Since the beginning of the pandemic, our institution created and mandated online COVID-19 training for all employees, which included content on transmission of the virus, identifying symptoms, healthy hygiene practices, and proper use of respirators and face coverings. 

**Table 1:** Characteristics of respondents.

	Respondents (n = 311)
Age	Average: 39 years
	Median: 38 years
	Range: 18 – 67 years
Gender	Female: 234 (76.5%)
	Male: 72 (23.5%)
Geographic location of training	West: 256 (84.2%)
	South: 33 (10.9%)
	Northeast: 9 (3%)
	Midwest: 6 (2%)

**Table 2:** Attitudes of PPE usage among responding healthcare workers. PPE: personal protective equipment; COVID-19: coronoavirus disease 2019

One year ago…	N (%)
How did you regard the importance of PPE?	Very important: 248 (79.74%)
	Somewhat important: 60 (19.29%)
	Not important: 3 (0.96%)
How frequently did you use PPE during a medical procedure that exposes you to a patient’s bodily fluids (i.e., biopsy, venipuncture, lavage)?	Always: 164 (53.59%)
	Often: 106 (34.64%)
	Sometimes: 31 (10.13%)
	Never: 5 (1.63%)
Currently…	
How do you regard the importance of PPE?	Very important: 289 (92.93%)
	Somewhat important: 20 (6.43%)
	Not important: 2 (0.64%)
How frequently do you use PPE during a medical procedure that exposes you to a patient’s bodily fluids (i.e., biopsy, venipuncture, lavage)?	Always: 245 (79.55%)
	Often: 49 (15.91%)
	Sometimes: 10 (3.25%)
	Never: 4 (1.30%)
What is the major influence on your perception of the importance of wearing PPE during procedures?	Recent pandemic (COVID-19): 138 (44.52%)
	Training/educational program: 106 (34.19%)
	Personal experience of splash to face: 39 (12.58%)
	Role modeling by faculty/colleagues: 20 (6.45%)
	Being observed by colleagues: 6 (1.94%)
	Residency program requirements: 1 (0.32%)

Questions regarding behaviors of respondents included both hygiene practices and frequency of wearing various PPE items (Table 3). Questions on hygiene practices showed that hand sanitization was “always” performed most frequently (73%) compared to cleaning shared spaces in the hospital (40%) and cleaning personal items (46%). Questions about PPE usage behaviors showed that respondents “always” wore the following: gloves (91%), face shield/mask with eye protection (35%), gown (41%), bouffant cap (34%), and shoe coverings (7.5%). Finally, 23% of respondents admitted suffering a splash to the face during a procedure while not wearing a face shield or the combination of both a mask and eye protection. 

**Table 3:** Behaviors of PPE usage among responding healthcare workers.

During procedures, how frequently do you wear…	N (%)
Gloves?	Always: 281 (91.23%)
	Often: 22 (7.14%)
	Sometimes: 4 (1.30%)
	Never: 1 (0.32%)
A gown?	Always: 126 (41.31%)
	Often: 116 (38.03%)
	Sometimes: 57 (18.69%)
	Never: 6 (1.97%)
Shoe coverings?	Always: 23 (7.47%)
	Often: 44 (14.29%)
	Sometimes: 123 (39.94%)
	Never: 118 (38.31%)
A bouffant cap?	Always: 104 (34.10%)
	Often: 69 (22.62%)
	Sometimes: 69 (22.62%)
	Never: 63 (20.66%)
A face shield or the combination of both mask and eye protection (glasses and goggles)?	Always: 108 (35.06%)
	Often: 132 (42.86%)
	Sometimes: 57 (18.51%)
	Never: 11 (3.57%)
How frequently do you…	
Sanitize your hands (using sanitizer or soap and water) before entering and exiting a patient’s room?	Always: 226 (73.38%)
	Often: 79 (25.65%)
	Sometimes: 2 (0.65%)
	Never: 1 (0.32%)
Clean shared spaces in the hospital (i.e., computer keyboards, table surfaces)?	Always: 124 (40.26%)
	Often: 141 (45.78%)
	Sometimes: 39 (12.66%)
	Never: 4 (1.30%)
Clean your personal items (i.e., cell phone, computer, and iPad screens)?	Always: 143 (46.28%)
	Often: 118 (38.19%)
	Sometimes: 46 (14.89%)
	Never: 2 (0.65%)
Have you ever suffered a splash to the face during a procedure while NOT wearing a face shield OR the combination of BOTH mask and eye protection?	Yes: 70 (22.73%)
	No: 238 (77.27%)

## Discussion

Strict adherence to PPE use among healthcare workers is crucial for preventing exposure to blood-borne infections and transmitting infections in the workplace; however, breaches in safety protocols are not uncommon. For example, data from the 2011 National Institute for Occupational Safety and Health (NIOSH) Health and Safety Practices Survey of Healthcare Workers showed a variable use of PPE among 1,800 nurses. Usage was greater when workers were more familiar with safe handling guidelines and the management commitment to safety was perceived to be higher [11]. Similarly, nearly a quarter of respondents in this study admitted to suffering a splash to the face in the absence of appropriate PPE. Another study found 26% of healthcare workers inappropriately touched the front of their mask while doffing, while approximately 50% touched a potentially contaminated PPE surface with an ungloved hand [12]. Comprehensive training that includes indications for PPE, correct identification of equipment, proper donning and doffing technique, and correct maintenance and disposal of PPE is critical for increasing the likelihood of using PPE when necessary.

Several factors may contribute to decreased compliance to workplace hygiene and safety standards, including insufficient education and training, organizational culture, discomfort with PPE use, and lack of access. A lack of knowledge on the importance of standard precautions as a fundamental countermeasure for infection transmission, for example, led to decreased adherence to PPE use among nurses in Japanese tertiary care hospitals [13]. Inadequate training procedures may also contribute to improper use of PPE; one study showed that contamination occurred in 79.2% of the PPE simulations, despite healthcare workers’ knowledge of being videotaped [14]. Additionally, a Cochrane systematic review found that poor workplace culture and lack of support from management negatively influenced healthcare workers’ attitudes towards PPE, and consequently, the desire to deliver quality patient care [15]. Discomfort caused by PPE, which may include headache from masks, fogging of eyewear, and skin irritation, may also discourage the use of PPE and the adherence to safety guidelines [16]. For example, one study evaluating equipment comfort and user attitude during the COVID-19 pandemic showed a significant positive correlation between the number of physical complaints and the subscale scores of participants' attitudes related to PPE (r= −0.004, p = 0.91 for protection subscale, r = 0.21, p = 0.001 for comfort and difficulty subscale, and r = −0.13, p = 0.001 for accessibility) [17].

Strategies for mitigating the barriers to PPE usage include comprehensive and effective education and training for healthcare workers, in addition to promoting a trusting and positive safety climate in the workplace. Arguments in support of national programs and standards for use of PPE have been made; currently, no such standardized program exists [18]. Education about infection precautions, indications for various elements of PPE gear, and correct donning and doffing techniques can help promote healthcare workers’ perception of the importance of PPE and adherence to safety guidelines [19]. This may be achieved by implementing standardized PPE protocols and devising innovative PPE education that effectively communicates and demonstrates specific safety handling procedures [11,14]. When healthcare workers are required to wear PPE for prolonged periods of time, such as during the COVID-19 pandemic, staffing considerations should plan for adequate periods of rest without wearing PPE in order to minimize discomfort [20].

The COVID-19 pandemic has far-reaching implications for the public’s attitudes toward PPE. Our survey results showed that the recent pandemic had the strongest impact on healthcare workers’ perception of the importance of wearing PPE (44%), even more so than educational/training programs (34%). This was corroborated by a shift in attitudes regarding the importance of PPE as very important (93%) compared to one year prior to survey dissemination, pre-pandemic (80%). While direct droplet spread is the major driver of infection due to COVID-19, hand hygiene and adherence to PPE protocol are just as paramount in combating viral transmission. Breaches in PPE protocol, hand-to-face touching, and surface contact are risks that can be mitigated by proper education which includes hands-on training, demonstrations, and observations by trained staff, as well as clearly outlining specific safety protocols respective to the institution to promote a positive and safe workplace climate.

Limitations of this study include results confined to a single institution, as well as non-response bias. Future studies that objectively quantify the impact of strategies for increasing PPE protocol adherence, such as through innovative educational/training programs, would be of interest for identifying solutions to improving PPE usage among healthcare personnel.

## Conclusions

Barriers to the use of PPE when it is indicated are multifactorial, and include inefficient education and training of healthcare staff, inadequate access to PPE, negative organizational culture, and discomfort associated with PPE use. The COVID-19 pandemic had a major impact on perceptions of the importance of PPE, as well as compliance with its use, in the efforts to mitigate infectious transmission.
